# Gig worker’s perceived algorithmic management, stress appraisal, and destructive deviant behavior

**DOI:** 10.1371/journal.pone.0294074

**Published:** 2023-11-08

**Authors:** Linzi Zhang, Jie Yang, Yiming Zhang, Guohu Xu

**Affiliations:** 1 School of Business Administration, Zhongnan University of Economics and Law, Wuhan, Hubei, China; 2 School of Management, South Central Minzu University, Wuhan, Hubei, China; Babes-Bolyai University, Cluj-Napoca, ROMANIA

## Abstract

With the advance of data technologies, gig platforms have applied data and algorithms to their management and put more stringent requirements on gig workers through algorithmic management. Gig workers might perform destructive deviant behavior when coping with algorithmic management. It is meaningful to examine how the algorithmic management applied to gig platforms could lead to gig workers’ destructive deviant behavior. Based on the challenge–hindrance framework, we developed a research model and validated it with survey data collected from 423 food delivery riders. We employed multi-level linear regression analysis in data analysis and found that perceived algorithmic management was appraised as both a hindrance and a challenge. As a hindrance, it elicits working/family deviant behavior; as a challenge, it helps reduce working/family deviant behavior. Regulatory focus (a prevention focus vs. a promotion focus) moderates the effect of perceived algorithmic management on stress appraisals (hindrance appraisals vs. challenge appraisals). This study explains algorithmic management’s impact on gig workers’ destructive deviant behavior through the appraisal of algorithmic management as both a challenge and a hindrance. It also provides practical advice to gig platforms, gig workers and policymakers on how to balance the challenge and hindrance roles of algorithmic management in gig work.

## 1 Introduction

The gig economy has broken barriers to labor mobility and helped workers share in the development dividends of the digital economy, and the platform-based gig economy is a popular form [1]. Gig platforms utilize algorithms to coordinate, surveil, and instruct gig workers in the case of decentralized working process and space [2]. This approach, called algorithmic management, enables platforms to guide gig workers to act in a manner consistent with platforms’ objectives. Ele.me, Uber, and Deliveroo are prime examples that use algorithmic systems to manage gig workers. Algorithms are used to assign tasks to, reward, or punish gig workers based on data obtained from these workers’ electronic devices [3].

The algorithmic management of gig workers could make them feel stressed about meeting a platform’s requirements and could even make them cope with this stress through destructive deviant behavior. For example, gig workers might run red lights or violate parking rules to complete their tasks within the time limits specified by gig platforms or to save their own time [4]. However, little knowledge has been obtained that explains how the algorithmic management of gig platforms could lead to gig workers’ destructive deviant behavior.

In the prior literature, algorithmic systems’ flexibility and autonomy have been widely discussed, and such systems’ roles in the continuous control and monitoring of workers have been highlighted. According to Jarrahi et al. [5], there is an “autonomy paradox.” On the one hand, unlike in traditional employment situations, where employees are required to adhere to strict commuting hours, working in a digital environment controlled by algorithms gives workers the flexibility and autonomy to choose when and where to work [6]. On the other hand, continuous tracking and monitoring via algorithmic systems might also trigger anxiety and tension among workers, making them feel less autonomous [7]. For instance, algorithmic management can be a source of stress for gig workers [6], which might result in irregular working hours, overworking, and social isolation for these workers [1]. Under platforms’ algorithmic management, gig workers feel unfairly treated and even express destructive deviant behavior. Although their destructive deviant behavior has attracted scholars’ attention, researchers have attempted few empirical studies to test how the algorithmic management of gig platforms could lead to gig workers’ destructive deviant behavior. Clearly, reducing destructive deviant behavior is important to help reduce organizational costs and improve organizational efficiency, as well as to improve gig workers’ work satisfaction and well-being. Therefore, exploring how platforms’ algorithmic management is associated with gig workers’ destructive deviant behavior is greatly valuable.

To fill this research gap, this study explores whether gig workers’ perceptions of algorithmic management lead to destructive deviant behavior, based on the challenge–hindrance framework. In prior research, scholars have argued that algorithmic management can be a positive stressor [8], while others have argued that it is a negative stressor [9]. However, it has been pointed out that classifying stressors a priori is unreasonable, and it is unclear whether work stress is a challenge or a hindrance [10]. Based on the transactional theory of stress, Kronenwett and Rigotti [11] pointed out that workers’ appraisals of stressors play a key role in determining different behavioral responses. Therefore, the challenge–hindrance framework was applied in this study to explain how algorithmic management leads to destructive deviant behavior among gig workers.

Additionally, stress appraisals vary among individuals due to their trait differences [10]. Regulatory focus (a prevention focus vs. a promotion focus) has been verified to be a crucial individual trait that affects individual appraisals of external stressors [12]. It is necessary to test whether prevention-focused and promotion-focused gig workers have different destructive deviant behaviors due to their different appraisals of the stress of algorithmic management. Therefore, this study tested the moderating role of gig workers’ regulatory focus in their appraisals of perceived algorithmic management, which could lead to their destructive deviant behavior.

This study contributes to the literature in three ways. First, algorithmic management was treated as a hindrance stressor or a challenge stressor a priori in prior research [8], however, this study emphasizes the vital role of individual stress appraisal and it explains algorithmic management’s impact on gig workers’ destructive deviant behavior by appraising the stressor of algorithmic management as both a challenge and a hindrance. Second, prior research has pointed out that platforms’ algorithmic management can trigger gig workers’ negative emotions [7], however, from the perspective of behavior, this study examines algorithmic management’s different roles as a challenge stressor and a hindrance stressor in leading to gig workers’ destructive deviant behavior both at and outside of work. Third, this study explains how gig workers’ individual traits (e.g., their regulatory focus) could affect their perceived algorithmic management as a challenge stressor and a hindrance stressor, which affect their destructive deviant behavior.

The remainder of this paper is structured as follows. The next section discusses the prior literature on gig platforms’ algorithmic management, workers’ destructive deviant behavior, the challenge–hindrance framework, and regulatory focus. Subsequently, the research model and hypotheses are proposed. Then, the research method is introduced, and the results of the study’s data analysis are reported. After our research findings are discussed, we highlight this study’s theoretical and practical implications, as well as its limitations, and we suggest avenues for future research.

## 2 Literature review

### 2.1 Research on gig platforms’ algorithmic management

In the management of gig platforms, a set of management rules are embedded into algorithms, making these platforms serve as automated managers [13]. The adoption of algorithms allows gig platforms to not only assign work to gig workers but also coordinate and direct their work and evaluate their work performance based on the real-time monitoring of their work process [14].

Algorithms that manage workers through automated decision-making with limited human interventions have been shown to generate profits for companies [5]. For example, as intermediaries connecting three parties (i.e., restaurants, delivery workers, and customers) in the delivery process, online delivery platforms manage workers’ performance through tracking mechanisms and customer ratings. This approach enables customers to become supervisors, managing workers’ job performance and, thus, keeping platforms’ labor costs low [15]. Additionally, it helps to improve customers’ trust [16] and intention to continuous use [17].

In previous research, scholars have also argued that algorithmic management can help organizations improve their management efficiency and achieve organizational goals, but they have ignored algorithmic management’s negative effects on workers [18]. Li [19] argued that platforms use algorithmic management as a tool to maximize labor exploitation, which undermines the gig economy’s promise to empower workers with autonomy and flexibility [1]. For workers, the decision-making mechanism of algorithmic management is opaque and constantly evolving [20], which may give rise to asymmetries in information and power [21]. As more critical decisions are made entirely by algorithms, workers are increasingly concerned about potential unfairness [22]. For example, faced with Uber’s algorithmic management, gig drivers generally complain about the constant monitoring, lack of transparency, and dehumanization of algorithmic management [23]. Algorithmic management could induce a sense of unfairness among workers, as well as low trust in their work [7] and low job satisfaction [21], ultimately reducing workers’ participation [24] without guaranteeing their work quality [25].

Though prior studies have found that algorithmic management may provoke negative emotions among gig workers, few studies have attempted to examine whether algorithmic management could lead to gig workers’ destructive deviant behavior. Therefore, in this study, we explore how gig workers respond to algorithmic management according to their destructive deviant behavior.

### 2.2 Destructive deviant behavior

Most of the extant literature on individual deviant behavior has focused on behavior in the workplace [26], and researchers have proposed two forms of deviant behavior, namely constructive and destructive deviant behavior. Specifically, constructive deviant behavior refers to the behavior through which employees take the initiative to violate organizational norms in order to improve the well-being of an organization or its members [9]. Destructive deviant behavior is a serious breach of organizational norms that hinders the well-being of an organization and its employees, constituting behavior directed against coworkers, supervisors, or the organization itself [27]. Additionally, Robinson and Bennett [27] pointed out that deviant behavior can be divided into organizational and interpersonal deviant behavior. They argued that the two types of deviant behavior can be distinguished according to whether the deviant behavior was directed against an organization or against its members. Later, Yam et al. [28] emphasized that employees may even transcend organizational boundaries and indulge in non-work-related deviant behavior, that is generally agreed to be undesirable. Individuals are likely to engage in deviant behavior that violates family norms, such as treating their parents unpleasantly, insulting their spouses, or harshly criticizing their children [29]. Lim and Tai [30] pointed out that family deviant behavior may violate the norms of mutual respect within a family and then escalate into uncivilized behavior.

Most of the extant literature has focused on deviant behavior’s negative effects. Chiu et al. [26] showed that employees’ deviant behavior negatively influences individual performance, even causing huge losses for an organization. The antecedents of employees’ deviant behavior mainly include factors within and beyond organizational control. The former includes organizational support and management styles [31]. The latter includes customer friendliness and role pressure; specifically, unfriendly customers may provoke workers’ destructive deviant behavior [32], and workers may also perceive role pressure as a hindrance to personal growth or something that prevents them from achieving their work goals, further inducing destructive deviant behavior [26].

Various work stresses have been verified to potentially trigger workers’ destructive deviant behavior, while the stress of platforms’ algorithmic management has been ignored in the literature. Furthermore, because the work and family spheres are integrated, the lack of a discussion of family deviant behavior may limit the understanding of work stress’s consequences. Therefore, in this study, we explored whether gig workers tend to perform working/family deviant behavior when facing the stress of algorithmic management at work.

### 2.3 The challenge–hindrance framework

The challenge–hindrance framework divides work stress into two categories: challenge stressors and hindrance stressors [33]. The former can be overcome through individual effort, while the latter impedes the individual achievement of goals [10]. A flaw in the challenge–hindrance framework is stressors are divided a priori into challenges and hindrances. However, the transactional theory of stress has pointed out that stressors are not the direct triggers of the stress response, and their influence on individuals largely depends on individual appraisals of stressors [34]. Individuals who believe stress will bring them rewards and growth appraise it as a challenge, while those who believe stress will harm their goal achievement and personal well-being appraise it as a hindrance [35]. Additionally, it has been confirmed that a stressor may be appraised as both a challenge and a hindrance, and the two are not mutually exclusive [11]. Webster et al. [36] studied work stressors and found that all stressors except for responsibility were positively correlated with both challenge appraisals and hindrance appraisals.

According to the transactional theory of stress, individuals’ challenge appraisals or hindrance appraisals influence their behavior in coping with stress [35]. These two appraisals inspire different coping behaviors [37]. On the one hand, when a stressor is appraised as a challenge, workers are motivated to put more effort into solving problems [38]. On the other hand, when a stressor is appraised as a hindrance, workers may distance themselves from the situation and reduce their work engagement [39]. Workers’ hindrance appraisals deplete self-resources to the point that they are unable to police their behavior, ultimately leading to uncivilized behavior toward others [11].

Therefore, based on the transactional theory of stress, in this study, we explore whether gig workers appraise platforms’ algorithmic management as both a challenge and a hindrance, ultimately inhibiting or increasing their destructive deviant behavior.

### 2.4 Regulatory focus

Lazarus and Folkman [35] asserted that individual traits interact with the external environment to jointly affect how stressors are appraised. Regulatory focus is a stable individual trait formed during an individual’s long-term growth, and it is important in understanding individual stress appraisals [12]. Higgins [40] proposed regulatory focus theory and pointed out that individuals have two self-regulatory systems, a prevention focus and a promotion focus, which explain individuals’ disparate coping strategies when they are in the same situation. Individuals with a prevention focus prefer strategies of precaution; they care about duties and stick to the “ought self.” In contrast, individuals with a promotion focus tend to adopt strategies of approaching desirable goals; they care about aspirations and the pursuit of the “idea self” [41].

Regulatory focus affects stress appraisals through uncertainty preferences and work emotions. In the uncertainty preference context, individuals with a promotion focus are generally open-minded at work, and they prefer variety to stability [42]. Conversely, individuals with a prevention focus keenly perceive uncertainty; they resist change and generally view changes as threats [43]. In the work emotion context, an individual with a promotion focus is usually highly self-efficacious; even if the task that individual is responsible for is difficult, they still have the confidence needed to complete it [44]. Instead, individuals with a prevention focus perceive organizational interventions as hindrances to their original work procedures, generating negative emotions, such as disappointment [45].

In this study, we discuss how gig workers with different regulatory focuses appraise the stress of algorithmic management.

## 3 Research model and hypotheses

**Fig 1:**
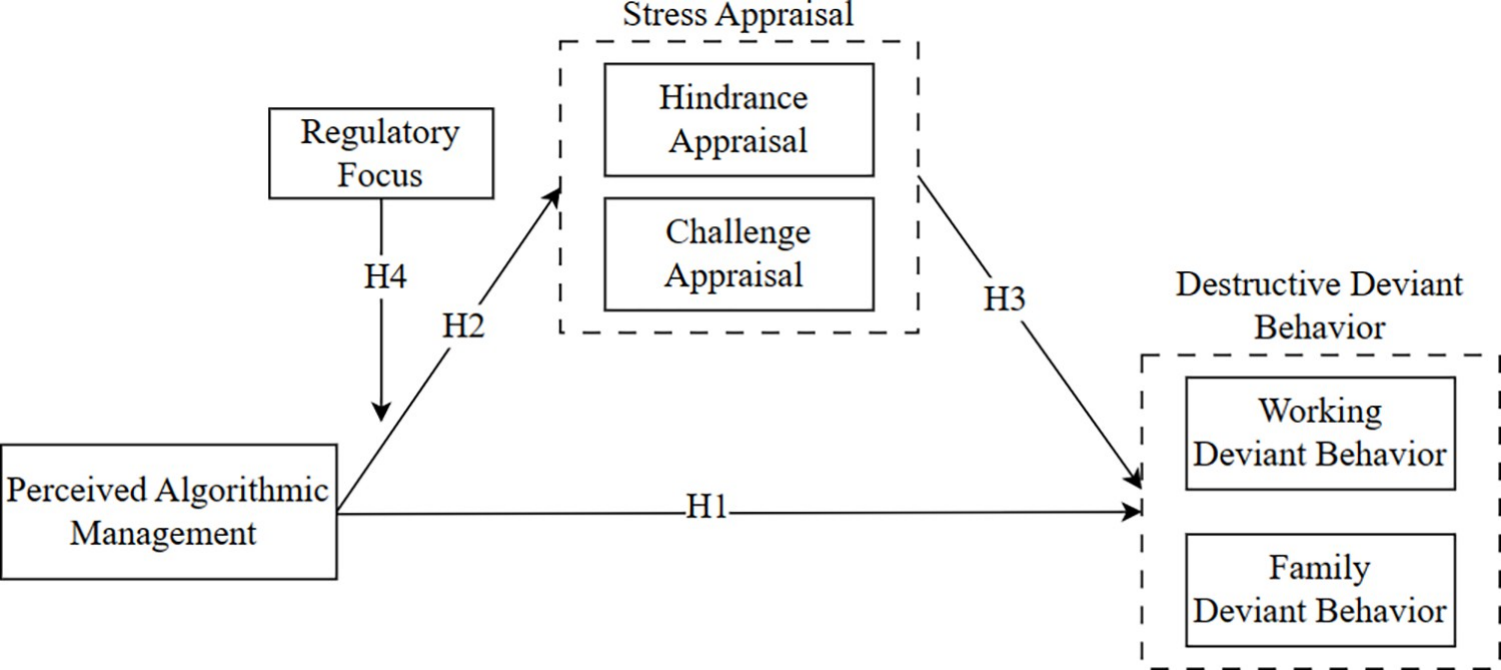
Research model.

In this study, we explored whether gig workers respond to the stress of perceived algorithmic management through destructive deviant behavior, including working deviant behavior and family deviant behavior. Specifically, based on the challenge–hindrance framework, we explored how gig workers’ stress appraisals of perceived algorithmic management as both a challenge and a hindrance, as well as their challenge or hindrance stress appraisal, affect their working/family deviant behavior. In addition, we tested how gig workers’ regulatory focus could moderate their stress appraisals of perceived algorithmic management as a challenge or a hindrance. Given that gig workers’ differences—such as gender, age, educational background, years of gig work, average monthly income, and working hours per day—may influence their destructive deviant behavior, the variables mentioned above were set as control variables. The research model is shown in Fig 1.

### 3.1 Gig workers’ perceived algorithmic management and destructive deviant behavior

To assign tasks and improve workers’ performance, gig platforms usually employ various algorithms that impose strict supervision on gig workers’ working process [24], which may give rise to physical and mental work stress among gig workers and, subsequently, stimulate them to perform destructive deviant behavior at work. Unlike traditional employees, who are fixed in particular departments, gig workers are treated as “independent contractors” by platforms [1], and their remuneration is mostly in the form of piecework [25]. To obtain a stable daily income, gig workers may have to devote time to work that should be spent with their families, which may provoke family deviant behavior. Therefore, we assumed that algorithmic management would influence not only gig workers’ working deviant behavior but also their family deviant behavior.

Chiu et al. [26] demonstrated that organizational and work factors are important antecedents of employees’ deviant behavior. In terms of organizational factors, deviant behavior can be attributed to an employee’s psychological contract violation and their sense of organizational injustice, both of which may be triggered by employees’ perceptions of a troubled employee–organization relationship [46]. Concerning work factors, Naseer et al. [47] asserted that the stress of task complexity and deadline requirements induce depression and ultimately give rise to deviant behavior among employees.

Gig platforms use algorithms to manage gig workers mainly in three ways: specification guidance, tracking evaluation, and behavioral constraints [48]. Platforms’ specification guidance based on algorithms reduces gig workers’ autonomy at work [49], which violates their original intentions to seek autonomous work. Because algorithms are too objective and ignore contextual factors [50], the continuous monitoring of gig workers’ working process by algorithms puts gig workers in a weak position due to tracking evaluation [51], leading to anxiety among workers [52]. As a behavioral constraint, platforms utilize reputation systems to manage gig workers [6] and transfer the right to supervise gig workers to customers so that gig workers appear to be under 24-hour supervision despite not being directly monitored by platforms [53]. Reputation systems also intensify competition among gig workers [54] and even produce the Matthew effect [55]. In other words, gig workers with higher ratings have access to more tasks; as a result, the salary gap between gig workers widens, which may aggravate gig workers’ perception of unfairness.

Individual negative emotions, such as anxiety, stress, and unfairness, require additional energy to adjust to, which depletes many mental resources [56], and then, behavioral reactions emerge after this resource loss [57]. Individuals with large resource losses are prone to behaving irrationally [58] and even expressing behavior that causes external harm so as to vent their negative emotions [59]. Additionally, individuals even bring work stress into their family lives [60]. Due to uncertainty concerning the time and number of tasks assigned by platforms, gig workers are often online all day to avoid missing any tasks [21]. High-intensity work consumes a huge amount of gig workers’ energy, which makes it difficult for them to handle family affairs, and they may even vent their work stress to their families. Hence, we proposed:

Hypothesis 1: Gig workers’ perceived algorithmic management positively influences their working deviant behavior and family deviant behavior.

### 3.2 The mediating effect of hindrance and challenge appraisal

According to the transactional theory of stress, it is not stressors but, rather, employees’ hindrance and challenge appraisals of stressors that trigger their reactions to stressors [61]. In the opinion of Lazarus and Folkman [35], employees’ appraisals of stressors can be divided into two stages: primary appraisals and secondary appraisals. The former determines how employees appraise external encounters, and the latter determines their behavioral strategies after appraisal [34]. Employees form hindrance appraisals when they think they cannot deal with stressors and may suffer losses, while they form challenge appraisals when they can handle stressors and gain rewards. It has been confirmed that hindrance and challenge appraisals are not mutually exclusive; in other words, a stressor can be appraised as a hindrance and a challenge simultaneously [62].

Comprehensive monitoring via platforms’ algorithms may cause a huge amount of stress among gig workers, and they, in turn, conduct a primary appraisal of this stress in the process of meeting algorithmic requirements. The service requirements demanded by algorithmic specification guidance increase gig workers’ difficulties with work [63]. For example, regulations requiring car-hailing service drivers to be polite and unable to cancel an order after receiving it bring a tremendous amount of stress to drivers [21], which may lead them to appraise algorithmic specification guidance as a hindrance to completing their work. As part of their tracking evaluations, platforms’ algorithms constantly track gig workers’ labor process and closely evaluate their service attitudes through electronic devices, and they set rule-based punishments according to situations of order refusal, unauthorized cancellation, and task timeouts [64]. This constant tracking and evaluation can result in anxiety among gig workers [65], which can be a hindrance to them. Algorithms impose behavioral constraints when platforms constrain gig workers through reputation systems [66], which may bring a sense of inequality and stress to gig workers [6]. In this case, algorithmic management may be appraised as a hindrance.

Most work requirements exert both negative and positive stressors on individuals [67]. Although gig workers may appraise platforms’ algorithmic management as a hindrance due to strict monitoring, they may also appraise it as a challenge because of the convenience provided by algorithmic management, such as providing personalized goals, technical support, real-time feedback, and incentive to gig workers. Studies on emotional responses to arousal conditions have confirmed that the human brain may experience negative and positive states simultaneously in response to the same stressor [68]. Therefore, it is plausible that gig workers may conduct both hindrance appraisals and challenge appraisals of algorithmic management.

As part of specification guidance, platform algorithms set personalized goals for each gig worker according to their portrait and then motivate them to improve their self-efficacy at work [14]. For example, Uber regularly sends personalized reports to drivers [69] to help them improve their ratings by making recommendations, such as suggesting that drivers provide free bottled water to passengers [70]. Moreover, by offering suggestions such as route planning to avoid traffic jams, platforms’ algorithms can help gig workers achieve their working goals and obtain rewards [71], so algorithmic specification guidance may be appraised by gig workers as a challenge to earn more income. Meanwhile, platforms use algorithms to assess gig workers’ work and then feed these assessments back to them in a timely manner to help improve their service quality [72]. Along this line of thinking, gig workers may appraise algorithmic management as a challenge in achieving their work goals. As a behavioral constraint, gig platforms set up rewards and punishments through algorithms to motivate gig workers to provide better services [18]. To obtain rewards such as cash and ratings, and to avoid punishments such as salary deductions, gig workers strive to regulate their behavior and then achieve their work goals [73]. The incentive mechanisms stimulate gig workers’ intrinsic motivation to earn more income and enhance their self-achievement through hard work [14], so behavioral constraints may serve as challenges for gig workers.

After a primary appraisal of perceived algorithmic management, gig workers conduct a secondary appraisal to decide how to cope with the stress created by algorithmic management. Individuals tend to engage in destructive deviant behavior with negative emotions when they appraise the result of stress from their job as a hindrance [35]. In light of the perspective mentioned above, gig workers are more likely to perform destructive deviant behavior when they regard algorithmic management as a stress that prevents them from accomplishing their work. Conversely, when individuals appraise job requirements as challenges, they believe their efforts at work will be rewarded, and then they explore solutions to work stress proactively [74]. In the same way, gig workers may reduce their destructive deviant behavior. Therefore, we proposed the following hypotheses.

Hypothesis 2: Gig workers’ perceived algorithmic management positively influence their hindrance appraisals and challenge appraisals.
Hypothesis 3: Gig workers’ stress appraisals significantly influence their destructive deviant behavior. Specifically, hindrance appraisals positively affect working/family deviant behavior, while challenge appraisals negatively affect working/family deviant behavior.

### 3.3 The moderating effect of regulatory focus

Promotion-focused workers and prevention-focused workers usually experience opposite emotions at work. According to regulatory focus theory, promotion-focused workers are more clearly oriented and intrinsically motivated, constantly improve their competence to achieve work goals, and usually experience positive emotions at work [44]. Therefore, promotion-focused gig workers prefer to appraise algorithmic management as a challenge and regard it as their responsibility to meet customers’ demands. Prevention-focused gig workers hold the view that platforms apply algorithms to intervene in their working process. In high-intervention working situations, they are more likely to experience negative emotions [75] and then appraise algorithmic management as a hindrance to completing their work. Take the drivers on online car-hailing platforms who are rated by passengers for example. They have to provide additional emotional labor to delight passengers [76], which may exacerbate the loss of their psychological resources, and then they tend to conduct hindrance appraisals.

Additionally, gig workers with a promotion focus and a prevention focus show a great difference in their preferences for uncertainty in work. With the help of algorithms, food delivery platforms set gamification mechanisms to encourage riders to complete more tasks [69]. Promotion-focused workers usually hold aggressive working attitudes toward uncertainty at work [77], and they tend to regard gamification mechanisms as challenges to increase their incomes. In this context, gig workers are more likely to associate algorithmic management with a challenging source of stress. However, prevention-focused workers are more concerned about uncertainty in their work [43], such as traffic accidents and extreme weather during deliveries, both of which dramatically increase the likelihood of delivery timeouts. Once a delivery time exceeds the standard set by algorithms, riders will receive fines from platforms or poor comments from customers. Hence, prevention-focused gig workers are more likely to view algorithmic management as a hindrance. Therefore, we proposed:

Hypothesis 4: Regulatory focus moderates the relationship between perceived algorithmic management and stress appraisals. Specifically, prevention-focused workers’ perceptions of algorithmic management have a stronger positive effect on hindrance appraisals, while promotion-focused workers’ perceptions of algorithmic management have a stronger positive effect on challenge appraisals.

## 4 Research method

### 4.1 Participants

We chose food delivery riders who work for online delivery platforms in China as our participants, and the reasons for this choice are as follows. First, food delivery riders are an important group in the world’s gig economy, and there are many workers in this group. Food delivery riders have become a crucial group that cannot be ignored in the development of the global gig economy. According to Lee et al. [78], by 2028, 90% of retail stores globally will meet customer demand through riders’ delivery services. Second, delivery riders interact closely with platforms’ algorithmic management, and their working process are always subject to algorithmic management. Based on these reasons, and considering the availability of data collection, in this study, we ultimately selected Chinese food delivery riders as research participants.

### 4.2 Procedure

Prior to data collection, this study obtained ethical approval from the academic committee of the first author’s home university. An online survey was applied in this study to collect data through the leading online survey platform Wenjuanxing (similar to Amazon’s Mechanical Turk) in China. Riders were informed of the objective of this study prior to participating and asked to sign an online informed consent form if they agreed to join in this study voluntarily. Then, the participants received a link to the questionnaire and proceeded to complete it online. It took about 10 minutes to complete the questionnaire. The online survey was conducted between June and July 2022. A total of 600 questionnaires were distributed, and 423 valid questionnaires were obtained after eliminating invalid responses, such as those from respondents who provided the same answers throughout the questionnaire. Table 1 presents the participants’ basic information.

**Table 1:** Participants demographics.

Items	Type	Number	Percentage
Gender	Male	288	68.1
	Female	135	31.9
Age	18–25	73	17.3
	26–35	144	34
	36–45	136	32.2
	≥46	70	16.5
Education	High school and below	163	38.5
	Junior college	136	32.2
	Bachelor	113	26.7
	Master	11	2.6
Work experience	≤1	189	44.7
	2–4	153	36.2
	5–7	50	11.8
	≥8	31	7.3
Average monthly income (CNY)	≤3000	45	10.6
	3001–5000	136	32.1
	5001–8000	216	51.1
	8001–10000	16	3.8
	≥10000	10	2.4
Daily working hours	≤2	19	4.5
	3–6	74	17.5
	7–10	220	52
	≥10	110	26
Full-time vs Part-time	Full-time	280	66.2
	Part-time	143	33.8

### 4.3 Measures

In this study, we applied validated construct items from the literature and adapted them to fit the research context of gig workers (e.g., food delivery riders). Specifically, we used items from the work of Pei et al. [48] to measure perceived algorithmic management, while the items used to measure hindrance appraisals and challenge appraisals were adapted from the works of LePine et al. [67] and Drach-Zahavy and Erez [79], respectively. The measurements of working deviant behavior and family deviant behavior were adapted from the works of Bennett and Robinson [29] and Li et al. [80], respectively. Promotion focuses and prevention focuses were measured using the items of Higgins et al. [81]. A five-point Likert scale (*1 = strongly disagree*, *5 = strongly agree*) was used to measure all construct items, and a complete list of the measurement items included in the model is attached in the S1 Appendix. The reliability and validity of each variable are shown in Table 2.

**Table 2:** Variable reliability and validity.

Variables	Items	Factor loading	Cronbach’s α	CR	AVE
Perceived algorithmic management (PAM)	PAM1	0.866	0.817	0.892	0.733
	PAM2	0.854			
	PAM3	0.848			
Hindrance appraisal (HA)	HA1	0.861	0.744	0.855	0.663
	HA2	0.761			
	HA3	0.817			
Challenge appraisal (CA)	CA1	0.882	0.882	0.919	0.739
	CA2	0.843			
	CA3	0.862			
	CA4	0.850			
Promotion focus (ProF)	ProF1	0.907	0.919	0.937	0.714
	ProF2	0.825			
	ProF3	0.792			
	ProF4	0.845			
	ProF5	0.854			
	ProF6	0.844			
Prevention focus (PreF)	PreF1	0.931	0.911	0.934	0.740
	PreF2	0.820			
	PreF3	0.880			
	PreF4	0.855			
	PreF5	0.808			
Working deviant behavior (WDB)	WDB1	0.704	0.772	0.855	0.597
	WDB2	0.787			
	WDB3	0.781			
	WDB4	0.814			
Family deviant behavior (FDB)	FDB1	0.843	0.822	0.884	0.655
	FDB2	0.791			
	FDB3	0.814			
	FDB4	0.789			

## 5 Data analysis and results

### 5.1 Confirmatory factor analysis results

We conducted confirmatory factor analysis to evaluate the structural validity between variables. The results in Table 3 show that the seven-factor model (χ2/df = 1.248, CFI = 0.989, TLI = 0.987, RMSEA = 0.024, SRMR = 0.034) had a better degree of fit than the alternative models, which indicated that the discriminant validity between the variables was better. To examine whether potential common method bias occurred, we added an additional unmeasured latent method factor to the seven-factor model, and the results showed that the fitting index of the model had not improved, indicating that there is no common method bias in our study.

**Table 3:** Structural validity.

Model	χ^2^	df	χ^2^/df	CFI	TLI	RMSEA	SRMR
1-factor model^a^	2951.068	377	7.828	0.668	0.642	0.127	0.152
2-factor model^b^	2663.511	376	7.084	0.705	0.681	0.120	0.148
3-factor model^c^	2318.196	374	6.198	0.749	0.727	0.111	0.141
4-factor model^d^	1788.877	371	4.822	0.817	0.800	0.095	0.134
5-factor model^e^	1250.586	367	3.408	0.886	0.874	0.075	0.112
6-factor model^f^	462.917	362	1.279	0.987	0.985	0.026	0.034
7-factor model^g^	444.279	356	1.248	0.989	0.987	0.024	0.034
7-factor with common method bias	466.543	355	1.314	0.986	0.984	0.027	0.043

^a^PAM+HA+CA+PreF+ProF+WDB+FDB

^b^PAM,HA+CA+PreF+ProF+WDB+FDB

^c^+PreF+ProF+WDB+FDB

^d^PAM,HA,CA,PreF+ProF+WDB+FDB

^e^PAM,HA,CA,PreF,ProF+WDB+FDB

^f^PAM,HA,CA,PreF,ProF,WDB+FDB

^g^PAM,HA,CA,PreF,ProF,WDB,FDB

Note: PAM: Perceived algorithmic management; HA: Hindrance appraisal; CA: Challenge appraisal; ProF: Promotion focus; PreF: Prevention focus; WDB: Working deviant behavior; FDB: Family deviant behavior.

As shown in Table 4, none of the relationship coefficients were greater than 0.75. Additionally, the values of the variance inflation factors (VIFs) ranged from 1.175 to 2.150, which was lower than 10 [82], indicating that there is no serious collinearity in this study.

**Table 4:** Correlations between variables.

Variable	PAM	HA	CA	WDB	FDB	ProF	PreF
PAM	1						
HA	0.199***	1					
CA	0.509***	-0.132**	1				
WDB	0.125**	0.552***	-0.230***	1			
FDB	0.165***	0.553***	-0.228***	0.656***	1		
ProF	-0.495***	0.136***	-0.576***	0.282***	0.275***	1	
PreF	0.533***	-0.112*	0.623***	-0.194***	-0.204***	-0.639***	1

Note

***p<0.001

**p<0.01

*p<0.05.

PAM: Perceived algorithmic management; HA: Hindrance appraisal; CA: Challenge appraisal; ProF: Promotion focus; PreF: Prevention focus; WDB: Working deviant behavior; FDB: Family deviant behavior.

### 5.2 Hypothesis testing

#### 5.2.1 The main effect and mediating effect

To test Hypothesis 1, we conducted multi-level linear regressions of destructive deviant behavior with perceived algorithmic management and control variables. Models 3 and 6 in Table 5 show that perceived algorithmic management has a significant positive effect on working deviant behavior (*β* = 0.154, *p* < 0.01) and family deviant behavior (*β* = 0.183, *p* < 0.001), and Hypothesis 1 was supported.

**Table 5:** Regression analysis.

Variable	HA	CA	WDB			FDB		
	Model 1	Model 2	Model 3	Model 4	Model 5	Model 6	Model 7	Model 8
Gender	0.049	-0.078	-0.135	-0.159*	-0.152	-0.111	-0.133	-0.128
Age	-0.007	-0.111*	0.068	0.072	0.044	0.075	0.078*	0.050
Education	-0.139**	0.088	-0.143**	-0.075	-0.124**	-0.153***	-0.090*	-0.133**
Working years	0.226***	-0.107*	0.228***	0.117**	0.204***	0.163***	0.061	0.139***
Average income	0.055	-0.170**	0.268***	0.242***	0.23***	0.276***	0.252***	0.238***
Working hours	0.002	0.034	0.084	0.083	0.091	0.019	0.018	0.026
PAM	0.249***	0.661***	0.154**	0.031	0.302***	0.183***	0.071	0.330***
HA				0.492***			0.449***	
CA					-0.224***			-0.223***
F	9.720***	28.726***	22.533***	41.864***	24.447***	22.572***	41.949***	25.505***
R	0.375	0.571	0.525	0.669	0.566	0.525	0.669	0.575
R^2^	0.141	0.326	0.275	0.447	0.321	0.276	0.448	0.330

Note: PAM: Perceived algorithmic management; HA: Hindrance appraisal; CA: Challenge appraisal; WDB: Working deviant behavior; FDB: Family deviant behavior.

Table 5 displays the effects of perceived algorithmic management on working/family deviant behavior and shows how hindrance appraisals and challenge appraisals mediate these effects. The results of Model 1 showed that the direct effect of perceived algorithmic management on hindrance appraisals was positive (*γ* = 0.249, *p* < 0.001), and models 4 and 7 respectively showed that the direct effect of hindrance appraisals on working deviant behavior (*γ* = 0.492, *p* < 0.001) and family deviant behavior (*γ* = 0.449, *p* < 0.001) were positive. Further, as shown in Table 6, the indirect effects of perceived algorithmic management on working deviant behavior (*ρ* = 0.123, 95% CI = [0.067, 0.185]) and family deviant behavior (*ρ* = 0.112, 95% CI = [0.060, 0.172]) through hindrance appraisals were significant, which were fully mediated, supporting hypotheses 2 and 3.

**Table 6:** The test of mediating effect.

	Total effect		Direct effect		Indirect effect			Mediation
	Effect	95%CI	Effect	95%CI		Effect	95%CI	
PAM- >WDB	0.154	[0.055, 0.253]	0.031	[-0.058, 0.120]	PAM->HA- >WDB	0.123	[0.067, 0.185]	Full mediation (indirect only)
			0.302	[0.191, 0.413]	PAM->CA- >WDB	-0.148	[-0.215, -0.086]	Partial mediation
PAM- >FDB	0.183	[0.092, 0.273]	0.071	[-0.011, 0.152]	PAM->HA- >FDB	0.112	[0.060, 0.172]	Full mediation (indirect only)
			0.330	[0.230, 0.431]	PAM->CA- >FDB	-0.147	[-0.205, -0.095]	Partial mediation

Note: PAM: Perceived algorithmic management; HA: Hindrance appraisal; CA: Challenge appraisal; WDB: Working deviant behavior; FDB: Family deviant behavior.

In Table 5, Model 2 shows that the direct effect of perceived algorithmic management on challenge appraisals was positive (*γ* = 0.661, *p* < 0.001), and models 5 and 8 respectively show that the direct effects of challenge appraisals on working deviant behavior (*γ* = −0.224, *p* < 0.001) and family deviant behavior (*γ* = −0.223, *p* < 0.001) were negative. Further, as shown in Table 6, the indirect effects of perceived algorithmic management on working deviant behavior (*ρ* = −0.148, 95% CI = [−0.215, −0.086]) and family deviant behavior (*ρ* = −0.147, 95% CI = [−0.205, −0.095]) through challenge appraisals were significant and partially mediated. Thus, hypotheses 2 and 3 were further supported.

#### 5.2.2 Moderation analysis

**Fig 2:**
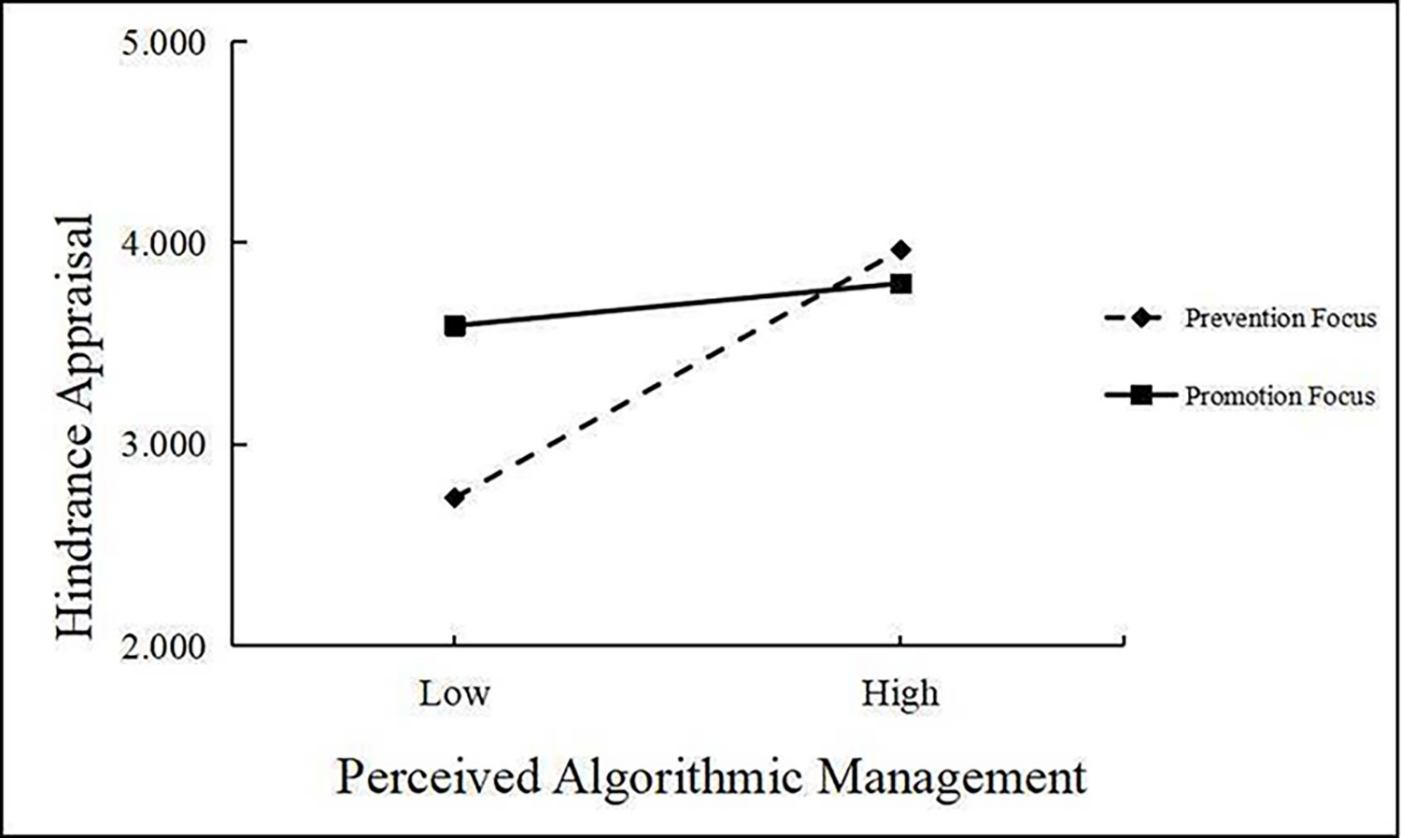
Interaction between PAM and regulatory focus on hindrance appraisal.

Next, we tested the moderating effects of regulatory focus on the relationship between perceived algorithmic management and working/family deviant behavior. As shown in Table 7, Model 2 revealed that the interaction between perceived algorithmic management and regulatory focus had a significant negative effect on hindrance appraisals (*γ* = −0.664, *p* < 0.001). It can be seen from Fig 2 that the effect of perceived algorithmic management on hindrance appraisals was stronger for prevention-focused gig workers than promotion-focused ones. Therefore, Hypothesis 4 was supported. Also, as illustrated in Table 8, the indirect effect of perceived algorithmic management on family deviant behavior through hindrance appraisals was stronger for prevention-focused gig workers (*ρ* = 0.359, 95% CI = [0.265, 0.457]) than promotion-focused ones (*ρ* = 0.061, 95% CI = [0.002, 0.123]). The difference in strength between these two effects was significant (*Δρ* = −0.298, 95% CI = [−0.400, −0.201]).

**Table 7:** The test of moderating effect.

Variable	HA		CA	
	Model 1	Model 2	Model 3	Model 4
constant	2.231***	3.307***	2.249***	4.220***
PAM	0.300***	0.482***	0.565***	0.340***
RF	0.233**	0.344***	-0.446***	-0.583***
PAM*RF		-0.664***		0.821***
F	9.574***	12.899***	29.189***	33.236***
R	0.395	0.468	0.601	0.648
R^2^	0.156	0.219	0.361	0.420

Note: PAM: Perceived algorithmic management; HA: Hindrance appraisal; CA: Challenge appraisal; RF: Regulatory focus.

**Table 8:** The test of moderated mediating effect.

Mediator	Moderator	Variable	WDB			FDB		
			Effect	SE	95%CI	Effect	SE	95%CI
HA	RF	ProF	0.067	0.034	[0.003,0.136]	0.061	0.031	[0.002,0.123]
		PreF	0.394	0.052	[0.294,0.498]	0.359	0.049	[0.265,0.457]
		Difference	-0.327	0.055	[-0.439,0.222]	-0.298	0.051	[-0.400,-0.201]
CA	RF	ProF	-0.172	0.040	[-0.253,-0.099]	-0.171	0.034	[-0.242,-0.108]
		PreF	0.012	0.015	[-0.017,0.044]	0.012	0.015	[-0.018,0.043]
		Difference	-0.184	0.046	[-0.281,-0.100]	-0.183	0.040	[-0.268,-0.111]

Note: PAM: Perceived algorithmic management; HA: Hindrance appraisal; CA: Challenge appraisal; ProF: Promotion focus; PreF: Prevention focus; WDB: Working deviant behavior; FDB: Family deviant behavior.

**Fig 3:**
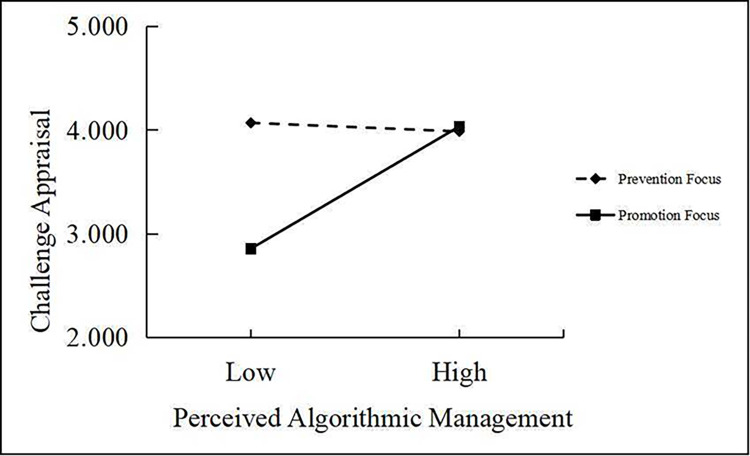
Interaction between PAM and regulatory focus on challenge appraisal.

In Table 7, Model 4 shows that the interaction between perceived algorithmic management and regulatory focus had a significant positive effect on challenge appraisals (*γ* = 0.821, *p* < 0.001). Fig 3 shows that the effect of perceived algorithmic management on challenge appraisals was stronger for promotion-focused gig workers than prevention-focused ones. Therefore, Hypothesis 4 was supported. Also, as illustrated in Table 8, promotion-focused gig workers’ perceived algorithmic management had stronger indirect effects through challenge appraisals on working deviant behavior (*ρ* = −0.172, 95% CI = [−0.253, −0.099]) and family deviant behavior (*ρ* = −0.171, 95% CI = [−0.242, −0.108]) than those of prevention-focused workers (*ρ* = 0.012, 95% CI = [−0.017, 0.044], and *ρ* = 0.012, 95% CI = [−0.018, 0.043], respectively). The differences in strength between the effects were significant (*Δρ* = −0.184, 95% CI = [−0.281, −0.100], and *Δρ* = −0.183, 95% CI = [−0.268, −0.111], respectively).

## 6 Discussions

First, through our research, we found that perceived algorithmic management provoke gig workers’ working/family deviant behavior. This finding is consistent with previous research; that is, the consumption of physical and psychological resources due to work stress provokes negative emotions, such as frustration, among workers [58], which leads workers to release stress through deviant behavior in order to reduce negative emotions [57]. Gig workers’ perceptions of algorithmic management create stress among them, and to preserve their existing resources, they may vent their negative emotions through working/family deviant behavior.

Second, as expected, we found that gig workers appraised perceived algorithmic management as both a challenge and a hindrance. Previous studies on workers’ appraisals of work stress have confirmed that the same stressor may be appraised as a challenge and a hindrance at the same time. Based on the challenge–hindrance framework, Gerich et al. [10] found that job demands were evaluated by workers as both challenge and hindrance stressors. Mitchell et al. [62] also pointed out that performance pressure is a double-edged sword that can be regarded as both a hindrance and a challenge by workers. On the one hand, algorithmic management can provide technical support for gig workers and help them make more rational decisions; thus, gig workers tended to appraise algorithmic management as a challenge. On the other hand, the constant monitoring and strict work requirements of algorithmic management can also trigger physical and mental stress among gig workers, and at this point, gig workers are more likely to appraise algorithmic management as a hindrance.

Third, we found that challenge appraisals negatively influenced working/family deviant behavior, while hindrance appraisals positively influenced working/family deviant behavior. Previous research has shown that workers’ challenge appraisals indicate that they believe they can handle a situation, which motivates them to be proactive in coping with stress, and this stimulating effect ultimately increases their engagement in their work [39]. Thus, when gig workers appraise algorithmic management as a challenge, they are less likely to engage in destructive deviant behavior. Conversely, hindrance appraisals indicate that workers believe they have no opportunity for growth and that their resources may not be able to be paid for; consequently, they tend to conserve resources to protect themselves from stress [11]. According to the conservation of resources theory, individuals tend to reduce their resource consumption by implementing negative behavior. Therefore, when gig workers appraise algorithmic management as a hindrance, they are more likely to engage in destructive deviant behavior.

Finally, our results revealed that gig workers’ appraisals of perceived algorithmic management are influenced by their regulatory focus. Specifically, promotion-focused workers are more likely to appraise perceived algorithmic management as a challenge. Previous research has demonstrated that promotion-focused individuals are committed to achieving goals and experience positive emotions [40]. Positive emotions help individuals maintain optimism and high self-efficacy [39]. Therefore, when perceiving algorithmic management, promotion-focused gig workers are more likely to appraise it as a challenge. Meanwhile, prevention-focused gig workers tend to appraise perceived algorithmic management as a hindrance. Prevention-focused workers are committed to avoiding risks and experience strong negative emotions [40], which leads them to perceive their work as meaningless. This lack of meaning in their work increases their sense of burnout at work, and they need to spend more energy to enhance their work’s meaning [44]. Thus, when perceiving algorithmic management, prevention-focused gig workers are more likely to appraise it as a hindrance.

## 7 Conclusions

### 7.1 Theoretical implications

Our study has three main theoretical implications. First, it extends the influence of algorithmic management from gig workers themselves to other groups, such as organizations, society, and families. Although the extant literature has demonstrated the negative effects of algorithmic management on gig workers themselves, such as their negative emotions and reduced work engagement, authors have rarely discussed whether gig workers’ behavior to cope with algorithmic management affects other groups. In this study, we have discussed whether gig workers’ coping behavior when facing algorithmic management adversely affects other groups at work (such as organizations and society), as well as family members outside of work. This discussion will enhance the understanding of gig workers’ behavioral responses to stress caused by platforms’ algorithmic management.

Second, this study highlights the role of gig workers’ stress appraisals in the research on the stress of algorithmic management, breaking through the traditional one-sided belief that algorithms are either harmful or beneficial. Most of the previous studies have directly classified algorithmic management as a challenge or a hindrance. However, in the context of gig workers’ stress appraisals, we proposed that whether algorithmic management is harmful or beneficial depends on workers’ stress appraisals, and it is worth noting that gig workers may appraise algorithmic management as both a challenge and a hindrance. This discovery expands the understanding of the role of algorithmic management as a challenge or a hindrance from the stress appraisal point of view.

Third, this study emphasizes that gig workers’ regulatory focus significantly influences their stress appraisals. Previous studies on gig workers’ stress appraisals have rarely taken their traits into account. The findings of the current study indicate that gig workers’ traits (e.g., regulatory focus) should be considered when examining their stress appraisals and coping with stress in their work. Our findings also enrich the understanding of gig workers’ stress appraisals of algorithmic management based on their traits.

### 7.2 Practical implications

In addition to its theoretical implications, our study also has practical implications for both gig workers, platforms, and policymakers. Gig workers can motivate themselves to shift from a prevention focus toward a promotion focus, which could help them appraise algorithm management as a challenge. For instance, they could take full advantage of the convenience provided by algorithms to complete their tasks if they are promotion-focused. Additionally, when gig workers cannot get the same rights as traditional employees (e.g., social security), a promotion focus can also help them actively invoke cognitive resources for self-guidance, understand the differences between platform employment and traditional employment, and take the initiative to adjust their emotions.

On the platform side, when applying algorithms to improve management efficiency and cut human resource costs, platform managers should be committed to fostering a harmonious relationship between gig workers and algorithms to promote the co-creation of their platform’s value. For instance, a gig platform should help gig workers understand the reason for algorithmic management and reduce their resistance to the algorithm. Additionally, in this study, we found that challenge appraisals can help reduce working/family deviant behavior, while hindrance appraisals have the opposite effect. Accordingly, gig platforms should introduce more humane modifications to algorithmic rules and improve gig workers’ positive understanding of the algorithm. Furthermore, policymakers could formulate regulations to guide gig platforms’ usage of algorithms in management, such as rules forbidding the use of algorithmic management for punishments and rewards of gig workers, disconnecting distribution time limits from delivery timeout punishments, and weakening incentive mechanisms (e.g., gamification) to reduce gig workers’ work stress, which could help decrease gig workers’ destructive deviant behavior and enhance their well-being in gig work.

### 7.3 Limitations and future research

Still, our study faced some limitations. First, we chose food delivery riders as our sample of gig workers; however, whether gig workers on other platforms, such as Uber and Rappi, appraise algorithmic management differently requires further discussion. Hence, we argue that, to improve the generalization of our research conclusions, the scope of this study’s research subjects could be further expanded in the future. Second, in our study, we defined gig workers’ perceptions of algorithmic management as part of the labor process, rather than in other forms, such as gatekeeping and guiding algorithmic management. Therefore, similar perspectives could be considered when defining this concept. Finally, there is intense competition in the Chinese job market, and most gig workers join a gig platform to make a living. However, due to differences in job markets and cultures, the purposes of joining a gig platform may differ in other countries. When gig workers’ purposes are to make more money or satisfy their personal interests, their appraisals of platforms’ algorithmic management and their tendency to engage in destructive deviant behavior may differ, so these aspects require further research in the future.

## Supporting information

S1 File Dataset. (ZIP)

S1 Appendix (DOCX)
