# Supplementary material for: Gig worker’s perceived algorithmic management, stress appraisal, and destructive deviant behavior
Source: PLoS One. 2023 Nov 8;18(11):e0294074. doi: 10.1371/journal.pone.0294074 (PMC10631696; doi:10.1371/journal.pone.0294074)
Supplement: S1 Appendix — (DOCX) [file pone.0294074.s002.docx]

# Appendix

| **Variables** | **Items** | **References** |
| --- | --- | --- |
| Perceived algorithmic management | **specification guidance:** | Pei et al., 2021 |
|  | 1. Algorithms set the target of delivery quantity for me. |  |
|  | 2. Algorithms assigns my tasks intelligently. |  |
|  | 3. Algorithms gives specification instructions to my work according to the standards by platform. |  |
|  | 4. Algorithms help me make decisions, such as analyzing road conditions and recommend the best route. |  |
|  | 5. Algorithms provide me with information, such as the number of regional orders. |  |
|  | 6. Algorithms gives me real-time feedbacks related to performance. |  |
|  | **tracking evaluation:** |  |
|  | 1. Algorithms tracks and locates my location in real time. |  |
|  | 2. Algorithms keeps track of my work. |  |
|  | 3. Algorithms monitors my work attitude in real time. |  |
|  | 4. Algorithms automatically evaluates the quality of my work. |  |
|  | **behavioral constraints:** |  |
|  | 1. Algorithms classifies my work performance and ranks it within platforms. |  |
|  | 2. Algorithms give priority to riders with higher service levels. |  |
|  | 3. Algorithms rewards me with cash at certain times to work hard. |  |
|  | 4. When I fail to meet a platform's requirements, algorithms fines me. |  |
| Hindrance appraisal | 1. Completing task hinders my happiness. | LePine et al., 2016 |
|  | 2. Job requirements limit my ability to achieve personal growth. |  |
|  | 3. My job is getting in the way of my personal achievement. |  |
| Challenge appraisal | 1. My job is a challenge to me. | Drach-Zahavy and Erez, 2002 |
|  | 2. My job helps me improve my ability. |  |
|  | 3. My job helps me overcome difficulties. |  |
|  | 4. My job helps me strengthen my self-esteem. |  |
| Promotion focus | 1. I always able to get what I want in my life. | Higgins et al., 2001 |
|  | 2. I always get my job done, even when it's hard. |  |
|  | 3. I often try to do different jobs well. |  |
|  | 4. I don't have to put too much effort into the things I love to do. |  |
|  | 5. I think I'm making progress in my job. |  |
|  | 6. I perform as well as I would have liked to have done on an important task. |  |
| Prevention focus | 1. I increase my job security by completing tasks. | Higgins et al., 2001 |
|  | 2. Security is very important to me when I look for a job. |  |
|  | 3. I take my duties very seriously at work. |  |
|  | 4. I think it's very important to do my job well. |  |
|  | 5. I do everything I can to reduce losses at work. |  |
| Working deviant behavior | 1. I swear and laugh at customers sometimes. | Bennett and Robinson, 2000 |
|  | 2. I conflict with vendors about the time of meal preparation. |  |
|  | 3. I deliberately tarnish the image of platforms through words and actions. |  |
|  | 4. I violate traffic regulations sometimes (driving through a red light, driving the wrong way, speeding, etc.) to ensure delivery timeliness. |  |
| Family deviant behavior | 1. I often vent negative emotions about work on family members. | Li et al., 2022 |
|  | 2. I often argue with my spouse because I can’t balance work and family. |  |
|  | 3. I often treat my parents impatiently because I spend too much energy in work. |  |
|  | 4. I often criticize my children harshly because of the high stress of work. |  |
